# Application of Distributed Parameter Model to Assessment of Glioma IDH Mutation Status by Dynamic Contrast-Enhanced Magnetic Resonance Imaging

**DOI:** 10.1155/2020/8843084

**Published:** 2020-11-22

**Authors:** Zongfang Li, Wei Zhao, Bo He, Tong San Koh, Yanxi Li, Yizhen Zeng, Zhuo Zhang, Jingzhong Zhang, Zujun Hou

**Affiliations:** ^1^Department of Radiology, The First Affiliated Hospital, Kunming Medical University, Kunming 650032, China; ^2^Department of Oncologic Imaging, National Cancer Center, Singapore 169610; ^3^Duke-NUS Graduate Medical School, Singapore 169857; ^4^Department of Anatomical & Cellular Pathology, The First Affiliated Hospital, Kunming Medical University, Kunming 650032, China; ^5^Institute for Infocomm Research, 1 Fusionopolis Way, #21-01 Connexis, Singapore 138632; ^6^Suzhou Institute of Biomedical Engineering and Technology, Chinese Academy of Sciences, Suzhou 215163, China; ^7^Zhengzhou Institute of Engineering and Technology Affiliated with SIBET, Zhengzhou 450001, China

## Abstract

Previous studies using contrast-enhanced imaging for glioma isocitrate dehydrogenase (IDH) mutation assessment showed promising yet inconsistent results, and this study attempts to explore this problem by using an advanced tracer kinetic model, the distributed parameter model (DP). Fifty-five patients with glioma examined using dynamic contrast-enhanced imaging sequence at a 3.0 T scanner were retrospectively reviewed. The imaging data were processed using DP, yielding the following parameters: blood flow *F*, permeability-surface area product PS, fractional volume of interstitial space *Ve*, fractional volume of intravascular space *Vp*, and extraction ratio *E*. The results were compared with the Tofts model. The Wilcoxon test and boxplot were utilized for assessment of differences of model parameters between IDH-mutant and IDH-wildtype gliomas. Spearman correlation *r* was employed to investigate the relationship between DP and Tofts parameters. Diagnostic performance was evaluated using receiver operating characteristic (ROC) curve analysis and quantified using the area under the ROC curve (AUC). Results showed that IDH-mutant gliomas were significantly lower in *F* (*P* = 0.018), PS (*P* < 0.001), *Vp* (*P* < 0.001), *E* (*P* < 0.001), and *Ve* (*P* = 0.002) than IDH-wildtype gliomas. In differentiating IDH-mutant and IDH-wildtype gliomas, *Vp* had the best performance (AUC = 0.92), and the AUCs of PS and *E* were 0.82 and 0.80, respectively. In comparison, Tofts parameters were lower in *K*^trans^ (*P* = 0.013) and *Ve* (*P* < 0.001) for IDH-mutant gliomas. No significant difference was observed in *K*ep (*P* = 0.525). The AUCs of *K*^trans^, *Ve*, and *K*ep were 0.69, 0.79, and 0.55, respectively. Tofts-derived *Ve* showed a strong correlation with DP-derived *Ve* (*r* > 0.9, *P* < 0.001). *K*^trans^ showed a weak correlation with *F* (*r* < 0.3, *P* > 0.16) and a very weak correlation with PS (*r* < 0.06, *P* > 0.8), both of which were not statistically significant. The findings by DP revealed a tissue environment with lower vascularity, lower vessel permeability, and lower blood flow in IDH-mutant than in IDH-wildtype gliomas, being hostile to cellular differentiation of oncogenic effects in IDH-mutated gliomas, which might help to explain the better outcomes in IDH-mutated glioma patients than in glioma patients of IDH-wildtype. The advantage of DP over Tofts in glioma DCE data analysis was demonstrated in terms of clearer elucidation of tissue microenvironment and better performance in IDH mutation assessment.

## 1. Introduction

As the most common primary tumor in the brain, diffuse glioma arises from the glial cells which provide support functions to neurons and presents with high morbidity and variable outcomes [1]. The 2016 World Health Organization (WHO) classification of tumors of the central nervous system included well-established molecular signatures, such as isocitrate dehydrogenase (IDH) mutation status, expression of the transcription regulator ATRX, and 1p/19q codeletion status [2], where IDH is a small molecule protein involved in a number of cellular processes, including mitochondrial oxidative phosphorylation, glutamine metabolism, lipogenesis, glucose sensing, and regulation of cellular redox status [3–5]. IDH gene mutation testing is an important prognostic biomarker in gliomas and is relevant for glioma patient management and glioma stratification [6, 7]. Previous studies showed that gene expression can significantly affect the disease course, and gliomas of IDH-wildtype appear to rapidly acquire multiple complex genetic alterations and become glioblastomas very early in their development, and glioma patients with mutant IDH had significantly longer overall survival than patients without IDH mutation [6–10]. The prognostic importance of IDH mutation is independent of other known prognostic factors, including age, grade, and MGMT methylation status [6]. Hence, IDH mutations could serve as an ideal target of therapy, and imaging parameters are highly potential to capture the biologic complexity underlying molecular phenotypes in gliomas.

However, conventional methods for assessment of IDH mutation status are through stereotactic biopsy invasive and prone to sampling error [11, 12]. Recently, noninvasive detection of IDH mutation status using functional imaging methods has received increasing attention [13–25]. In addition to tissue anatomic structure, functional imaging measures tissue microenvironment and provides in vivo physiologic information about brain tumors. An important functional imaging method is contrast-enhanced magnetic resonance imaging (MRI), which includes T1-weighted dynamic contrast-enhanced imaging (DCE) and T2-weighted dynamic susceptibility contrast-enhanced imaging (DSC), both of which have been applied to IDH mutation assessment in gliomas [20–25].

The tissue microenvironment of frequent study includes tumor vascularity and vessel permeability. The former is modeled as cerebral blood volume (CBV) in DSC and plasma fractional volume (*Vp*) in DCE. The value of CBV is often normalized with respect to a reference tissue, as denoted by relative CBV (rCBV) or normalized CBV (nCBV). Among existing studies using DSC or DCE for IDH mutation status assessment, discrepancies between different studies were evident. A significantly higher rCBV in IDH-wildtype compared with IDH-mutant type gliomas in all histological grades was reported in [20], whereas rCBV between IDH-wildtype and IDH-mutant gliomas did not differ significantly in histological subtypes of astrocytomas and oligodendrogliomas in [21]. Tissue vascularity was found to be significantly higher in IDH-wildtype gliomas than in IDH-mutant gliomas using DSC in [23]. However, tissue microenvironment parameters showed no correlates with glioma IDH mutation status using DCE in [24, 25] or DSC in [25].

The apparent conflicting results could be due to difference in imaging protocol, patient cohort, or tracer kinetic models for analyzing the acquired contrast-enhanced imaging data. Existing studies mostly utilized conventional tracer kinetic models such as the Tofts or extended Tofts model [26, 27], which does not differentiate the intravascular transport of tracer molecular with respect to the exchange process of tracer molecular between intravascular and interstitial spaces. There has been progress in the development of more advanced techniques in analyzing DCE data, such as the conventional compartment model (CC) [28], the adiabatic approximated tissue homogeneity model (ATH) [29], and the distributed parameter model (DP) [30]. The aforementioned two transports were separately accounted in these models, where blood flow (F) is utilized to characterize the intravascular transport and permeability-surface area product (PS) to describe the exchange between intravascular and interstitial spaces. In comparison, these two transports are modeled using one parameter, transfer constant (*K*^trans^), in the Tofts or extended Tofts model. Interested readers could refer to [31, 32] for a review on the topic.

So far, few studies have been carried out on the investigation of these advanced tracer kinetic models in glioma molecular subtype characterization. Because advanced tracer kinetic models provide more realistic description of tracer transport in tissue microenvironment, it is expected that the derived parameters could be more interpretable with respect to tumor tissue microenvironment. This study hypothesizes that IDH mutations reduce the enzymatic activity of the encoded protein [7], leading to change in tissue microenvironment, and parameters derived using advanced tracer kinetic models would be more closely associated with glioma molecular signatures. Using DP as example, this study attempts to explore its application to glioma IDH mutation differentiation.

## 2. Materials and Methods

### 2.1. Subjects

**Figure 1:**
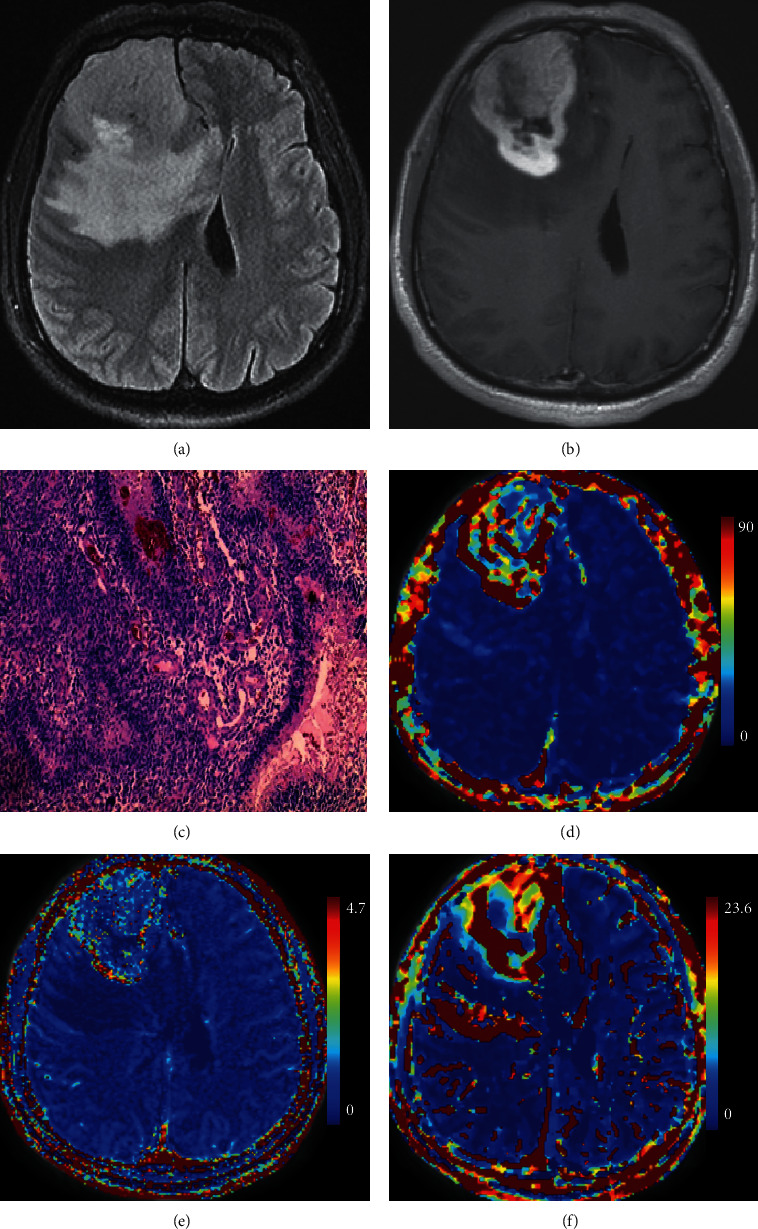
A 54-year-old male with IDH-mutant glioblastoma in the right front lobe. (a) FLAIR, showing a mass of slight hyperintensity with surrounding high signal edema in the right front lobe; (b) contrast-enhanced T_1_WI, showing a heterogeneous contrast-enhanced mass with irregular shape; (c) histopathological image of hematoxylin-eosin stain (x10); (d) permeability-surface area product PS (mL/min/100 mL); (e) fractional volume of the intravascular space *Vp* (mL/100 mL); (f) fractional volume of the extravascular extracellular space *Ve* (mL/100 mL) by the distributed parameter model.

This retrospective study was approved by the institutional review board. Sixty-one patients were included in this study between August 2017 and September 2019. All patients diagnosed with gliomas of grade II–IV according to the 2016 WHO guideline on brain tumor classification after craniotomy and tumor resection. Patients in the study did not have a history of previous surgery for brain tumor. Six patients were excluded due to inadequate MRI quality. A total of 55 patients (23 men, 32 women; age range, 25–72 years; mean age, 46.45 ± 10.23 years) were included in the study. There were 7 oligodendrogliomas (WHO grade II), 11 astrocytomas (WHO grade II), 2 anaplastic oligodendrogliomas (WHO grade III), 8 anaplastic astrocytomas (WHO grade III), and 27 glioblastomas (WHO grade IV). Molecular pathological findings of IDH were determined by Sanger sequencing for IDH hotspot mutations. There were 24 patients with IDH mutation. A representative case is given in Figure 1.

### 2.2. MR Imaging Acquisition

All scans were performed using a 3.0 T MRI scanner (Discovery MR750w, GE Healthcare, Milwaukee, USA). Anatomical scans included axial T1-weighted imaging (T_1_WI), T2-weighed imaging (T_2_WI), and fluid-attenuated inversion recovery (FLAIR), with parameters as follows. T1WI: repetition time (TR)/echo time (TE)/inversion time (TI) (2182 ms/22.7 ms/753 ms), field of view (FOV) (220 × 220 mm^2^), matrix (320 × 256), and slice thickness (5 mm); T2WI: TR/TE (4879 ms/116 ms), FOV (220 × 220 mm^2^), matrix (416 × 416), and slice thickness (5 mm); and FLAIR: TR/TE/TI (9000 ms/94 ms/2474 ms), FOV (220 × 220 mm^2^), matrix (256 × 256), and slice thickness (5 mm). DCE scan was performed using an axial fast-spoiled gradient (SPGR) echo sequence, with a precontrast and a postcontrast sequence: TR/TE (5.318 ms/1.196 ms), FOV (240 × 240 mm^2^), matrix (256 × 192), slice thickness (5 mm), flip angles of precontrast scan (4°, 8°, and 15°), and postcontrast scan (15°). Ten dynamic precontrast scans were acquired for each flip angle, and the postcontrast sequence consisted of 180 dynamic scans, with temporal resolution 2 seconds. The contrast agent was Gadovist (Magnevist; Bayer Schering Pharma AG) at an injection rate of 3 mL/sec (followed by a 20 mL normal saline flush) and a dose of 0.1 mmol/kg body weight.

### 2.3. Image Processing

All image processing was performed using a commercially available software (MItalytics, FITPU Healthcare, Singapore), where the method of the variable flip angle was utilized for estimating tissue contrast concentration. Voxel-wise fitting using Tofts and DP was applied to the concentration-time curve as derived from DCE data, yielding the following kinetic parameters: transfer constant *K*^trans^ (min^−1^), fractional volume of extravascular extracellular space *Ve* (mL/100 mL), efflux rate constant *K*ep (min^−1^, which is actually derived as the ratio between *K*^trans^ and *Ve*) in Tofts, and fractional volume of intravascular space *Vp* (mL/100 mL), blood flow *F* (mL/min/100 mL), permeability-surface area product PS (mL/min/100 mL), extraction ratio of first pass *E* (%), and *Ve* in DP. Tumor region of interest (ROI) was manually delineated by an experienced neuroradiologist (20 years' experience) with cross-referencing anatomical imaging, while blinded to patients' pathology results. Tumor segmentation includes both the nonenhancing and the enhancing tumor core, but obvious necrosis, cystic change, hemorrhage, large vessels, and definite perilesional edema were avoided. For completeness, the Tofts and the DP models were briefly described in the following.

Tofts can be modeled [26] by

(1) $$\begin{array}{c} C_{\text{tiss}}\left(t\right)=C_{A}\left(t\right)⊗K^{\text{trans}}\exp\left(-\frac{K^{\text{ttrans}}}{Ve}t\right), \end{array}$$

where *C*_tiss_ (*t*) represents the tissue tracer concentration-time curve, *C*_A_ (*t*) denotes the concentration of tracer in blood plasma in a feeding artery (also called the arterial input function AIF), and ⊗ denotes the convolution operator.

The distributed parameter (DP) model [30] accounts for concentration gradients in the vascular and interstitial compartments, with *C*_tiss_ (*t*) given by

(2) $$\begin{array}{c} C_{\text{tiss}}\left(t\right)=C_{A}\left(t\right)⊗,\\ F\left[\begin{array}{c} u\left(t\right)-u\left(t-\frac{Vp}{F}\right)+\\ u\left(t-\frac{Vp}{F}\right)\left\{1-\exp\left(-\frac{\text{PS}}{F}\right)\left[1+\overset{t}{\underset{0}{\int }}\exp\left(-\frac{\text{PS}}{Ve}\tau \right)\sqrt{\frac{\text{PS}}{Ve}\frac{\text{PS}}{F}\frac{1}{\tau }}I_{1}\left(2\sqrt{\frac{\text{PS}}{Ve}\frac{\text{PS}}{F}\tau }\right)d\tau \right]\right\} \end{array}\right], \end{array}$$

where *I*_1_ is the modified Bessel function.

The most remarkable difference between two models lies in the description of two transports of tracer molecular in tissue microenvironment, namely, the intravascular transport and the exchange between the intravascular space and the interstitial space. DP separately accounts for these two processes and uses *F* to describe the former transport and PS to characterize the latter, whereas Tofts mixes two processes and use *K*^trans^ to model.

The precision of the model parameters under different noise conditions has been studied using the Monte Carlo simulation approach in [30, 33, 34]. The level of uncertainty (or error) associated with the estimation of the model parameter was assessed using the coefficient of variation (CV). Using Monte Carlo simulation in various noisy conditions, CVs of estimated parameters are generally small. For the tumor, the CV values for all parameters improve to a more acceptable level of about 13% or less for a signal-noise-ratio (SNR) of 20, where SNR is taken to be the ratio of the maximum value of *C*_tiss_ (*t*) (before noise was added) and the standard deviation of noise.

### 2.4. Statistical Analysis

Tumor voxels of each patient were pooled together, and median values of kinetic parameters were calculated. Statistical calculations were performed using R software (version 3.6.1). The normal distribution was tested by the Shapiro–Wilk test. A Wilcoxon test was performed to compare the medians of kinetic parameters between IDH-mutant and IDH-wildtype groups. Boxplots were employed for displaying the data dispersion and for visual comparison on the distribution of kinetic parameters in IDH-mutant and IDH-wildtype groups. Statistical significance was set at *P* < 0.05. The receiver operating characteristic (ROC) curves were utilized for assessing the performance of kinetic parameters in differentiating IDH-mutant from IDH-wildtype gliomas. The diagnostic performance was quantified using the area under the ROC curve (AUC). The method of Youden index was utilized for computing the optimal threshold and the corresponding sensitivity and specificity, where the optimal threshold is selected as the one to maximize the sum of sensitivity and specificity [35]. Relationship between Tofts and DP model parameters was assessed using the Spearman correlation test.

## 3. Results

### 3.1. Median Values of Kinetic Parameters

**Figure 2:**
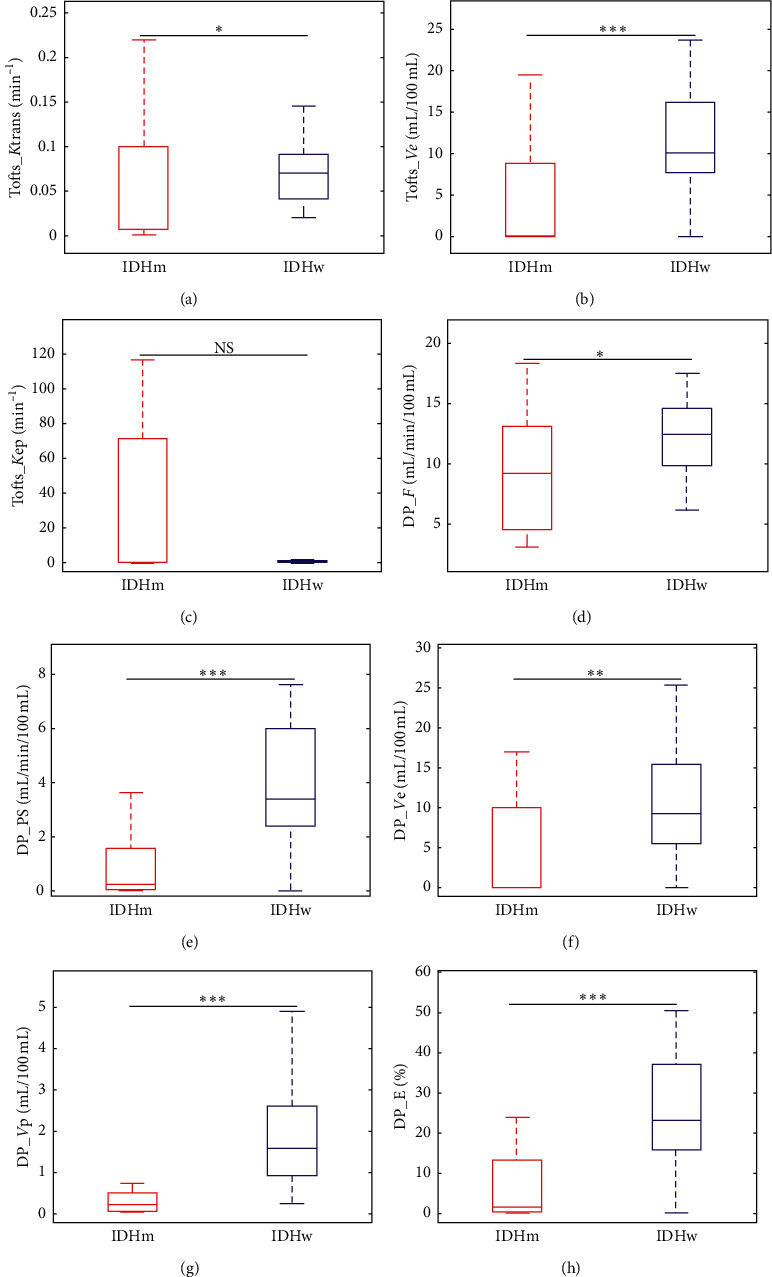
Boxplots of kinetic parameters in differentiating IDH-mutant (IDHm) with wildtype (IDHw) of gliomas, where (a)–(c) are Tofts parameters *K*^trans^, *Ve*, and Kep; (d)–(h) are DP parameters *F*, PS, *Ve*, *Vp*, and *E*, respectively. NS stands for not significant. ^*∗*^*p* < 0.05, ^*∗∗*^*p* < 0.01, and ^*∗∗∗*^*p* < 0.001.

Table 1 summarizes the median values of kinetic parameters of two models in IDH-mutant gliomas and IDH-wildtype gliomas, the interquartile range (namely, the range between the 25^th^ and the 75^th^ percentile), as well as the results of the Wilcoxon test. The values of *K*^trans^ and PS in IDH-mutant gliomas were smaller than in IDH-wildtype gliomas, and the difference in *K*^trans^ (*P* = 0.013) was less significant than in PS (*P* < 0.001). The values of *Ve* in both models in IDH-mutant gliomas are significantly smaller than in IDH-wildtype gliomas (*P* < 0.001 for Tofts and 0.002 for DP). The values of *Vp* and *E* in IDH-mutant gliomas were significantly lower than in IDH-wildtype gliomas (*P* < 0.001). Comparatively, the difference in the values of *F* between IDH-mutant and IDH-wildtype gliomas was less significant (*P* = 0.018). The difference in Tofts-derived *K*ep was not statistically significant (*P* = 0.525).  Figure 2 shows the boxplots of DP and Tofts parameters in differentiating IDH-mutant and IDH-wildtype gliomas. The distribution of all data was not normal using the Shapiro–Wilk test at the 5% significance level. It was of note that though a few parameters (like Tofts-derived *Ve*, DP-derived PS, *Ve*, *Vp*, and *E*) showed a very significant difference between IDH-mutant and IDH-wildtype gliomas at the 0.1% significance level; only the distributions of DP-derived *Vp* in IDH-mutant and IDH-wildtype gliomas were well separated. The large difference in data dispersion of Tofts-derived *K*ep between IDH-mutant and IDH-wildtype gliomas indicated low reliability of this estimated parameter.

### 3.2. ROC Curve Analysis

**Figure 3:**
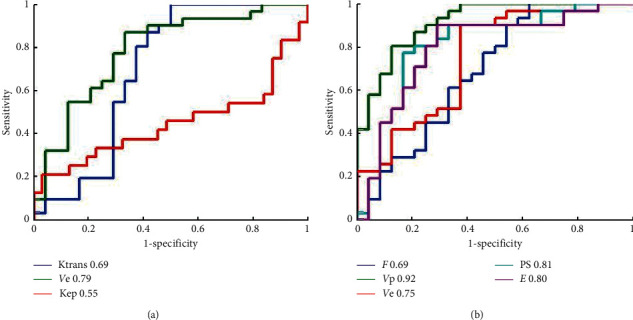
Receiver operating characteristic (ROC) plots and areas under ROC curve (AUCs) of (a) Tofts and (b) DP model parameters in differentiating IDH-mutant with IDH-wildtype of gliomas.

Table 2 summarizes the results of ROC curve analysis in differentiating IDH-mutant from IDH-wildtype gliomas. DP-derived *Vp* attained the best performance in discriminating IDH-mutant from IDH-wildtype gliomas (AUC = 0.92), yielding sensitivity 87.5% and specificity 80.7% with cutoff value 0.74. Two permeability parameters PS and *E* also showed promising discriminative power (AUC = 0.81 and 0.80, respectively). Among Tofts-derived parameters, *Ve* showed the best performance in IDH mutation type differentiation with AUC 0.79. The AUCs of *K*^trans^ and *K*ep were 0.69 and 0.55, respectively. The plots of ROC curves are shown in Figure 3.

Table 3 summarizes the correlation coefficients (*r*) between Tofts and DP model parameters in IDH-mutant and IDH-wildtype gliomas using the Spearman correlation test, where the value in bracket was the *P* value of the test. Of first note was the not statistically significant correlation for Tofts-derived *K*^trans^ with all DP-derived parameters, and in particular, the weak correlation between *K*^trans^ and *F* (*r* = 0.29, *P* = 0.168 in the IDH-mutant group; *r* = 0.24, *P* = 0.194 in the IDH-wildtype group) and the very weak correlation between *K*^trans^ and PS (*r* < 0.01, *P* = 0.982 in the IDH-mutant group; *r* = 0.05, *P* = 0.808 in the IDH-wildtype group). Tofts-derived *Ve* showed a very strong correlation with DP-derived *Ve* in either IDH-mutant or IDH-wildtype gliomas (*r* ≥ 0.94, *P* < 0.001), and showed a moderate to strong correlation with most other DP-derived parameters, in particular in IDH-mutant gliomas with respect to *Vp*, PS, and *E* (*r* > 0.75, *P* < 0.001).

## 4. Discussion

In this study, state-of-the-arts in DCE MRI tracer kinetic modeling were applied to the differentiation of IDH-mutant from IDH-wildtype gliomas. The former was found to be characterized with lower blood flow, lower permeability, lower *Vp*, and lower *Ve*. The advanced tracer kinetic modeling technique was compared with the conventional Tofts model. It turned out that DP largely outperformed Tofts in IDH-mutant and IDH-wildtype differentiation.

*Vp* was found to be the most distinct feature in discriminating IDH-mutant from IDH-wildtype gliomas, with a significantly lower value in the IDH-mutant group (median, 0.21; interquartile range, 0.06–0.44) than in the IDH-wildtype group (median, 1.59; interquartile range, 0.93–2.43). Gliomas are characterized by extreme tortuosity in their capillary bed [36]. In the early stages, tumor hypoxia leads to increased expression of hypoxia-inducible factor-1*α* (HIF-1*α*), which in turn mediates an increase in the vascular endothelial growth factor/vascular permeability factor (VEGF/VPF) [37, 38] that stimulates the growth of new, immature, leaky blood vessels (neovascularization). As these vessels mature, vascular intussusception and vascular cooption occurs, resulting in increased microvascular density [38, 39]. In DCE tracer kinetic modeling, *Vp* measures the fractional volume of the intravascular space, which could be linked to the density of tissue microvasculature and is in the concept similar to CBV in the tracer kinetic modeling of DSC data. In this sense, the finding is consistent with previous studies [23, 40] which demonstrated that IDH-mutant tumors showed a significantly lower rCBV as compared to the wildtype counterpart. The significantly decreased *Vp* from IDH-wildtype to IDH-mutant in Table 1 suggests that IDH-mutant gliomas correspond to reduced microvascular density.

In advanced tracer kinetic modeling, two types of tracer transport in tissue microenvironment are accounted for intravascular perfusion and exchange between intravascular space and extravascular space, which are explicitly described and separately characterized by the blood flow (*F*) and permeability-surface area product (PS). *E* estimates the ratio of tracer leakage during the first pass and represents another permeability parameter. As shown in Table 1, the difference in *F* between IDH-mutant and IDH-wildtype gliomas is evidently less significant than the difference in PS and *E* (*P* = 0.018 vs *P*<0.001). As pointed out in [39, 41], blood flow can be extremely variable and heterogeneous in any given region of a tumor. The diagnostic power of *F* is inferior to that of PS and *E* in differentiating IDH-mutant from IDH-wildtype gliomas (AUC = 0.69 vs 0.81, 0.80).

The increased PS and *E* from IDH-mutant to IDH-wildtype indicate the increased leakiness of vessels in IDH-wildtype gliomas. The leakiness of blood vessel could be affected by multiple factors, such as the luminal surface area, permeability of the vessel wall, blood flow, and hydrostatic, interstitial, and osmotic gradients across the endothelium [41–43]. To ensure the delayed permeability is measured, the postcontrast scan in this study captures not only the first pass of the contrast agent but also the bidirectional exchange between the intravascular space and interstitial space, which lasts about 6 mins in total. Interested readers can refer to [44, 45] for analysis of tracer transport and imaging protocol design. The good performance of PS in this study illustrates the prominent feature of leaky vessels in gliomas and justifies the separate modeling of intravascular transport and bidirectional exchange between intravascular space and extravascular space in advanced tracer kinetic modeling techniques.

Parameter *Ve* can be interpreted as the fractional volume of the extravascular extracellular space, which may reflect mitotic activity of cells. Tumor tissue is typically characterized by overgrowth of tumor cells, leading to the decreased interstitial space. In general, the higher the cells' mitotic activity, the smaller the interstitial space. Nevertheless, care should be taken in the interrogation of *Ve* in brain tumor. In our study, evident increase in *Ve* was observed in IDH-wildtype gliomas (*P* = 0.002 for DP), which is inconsistent with previous studies [24, 25], where no significant difference was observed on *Ve* values between IDH-mutant and IDH-wildtype gliomas. This might be due to the differences in imaging protocols and patient demography. The temporal resolution is 4 ∼ 5 seconds in [25], and the study by [24] focused on high-grade glioma with postcontrast scan time about 2 mins, which primarily captures the first pass of contrast transport. This study was performed on both low-grade and high-grade gliomas.

Nevertheless, the finding of higher *Ve* did not mean that IDH-wildtype gliomas had lower mitotic activity of cells. The apparent controversy could be explained as follows. Compared with other tumors (like liver tumor), vessel permeability is much smaller in brain tumor due to the presence of the blood-brain barrier (BBB). The exchange between intravascular space and interstitial space is a very slow process in brain tumor due to the presence of BBB. In particular, to measure the compromise of BBB using DCE imaging, scanning time is usually much longer than in tumor imaging, typically 20∼30 minutes [46]. As such, the insufficient scan time only allows to observe the exchange process during this period and to measure the actual volume in the interstitial space as manifested by the contrast agent during this period. This “measured” interstitial space closely reflected the vessel permeability. Studies using DWI found that IDH-wildtype gliomas had lower apparent diffusion coefficient than IDH-mutant gliomas, indicating more restricted diffusion space of water molecular in IDH-wildtype gliomas [21]. Thus, the “actual” extravascular extracellular space in IDH-wildtype gliomas would be smaller than that in IDH-mutant gliomas. Therefore, different from applications to most other types of solid tumor, the “measured” *Ve* in glioma DCE did not tell the mitotic activity of tumor cells, and the increased *Ve* in IDH-wildtype gliomas might indicate elevated vessel permeability in these gliomas.

This seems to be in contrast with the recent finding by Mills and colleagues [47] on glioblastoma, which shows that high values of *Ve* are significantly associated with increased mitotic activity. Nevertheless, Mills and colleagues [47] also stated that the positive correlation between *Ve* and mitotic activity was “unexpected.” Furthermore, it is pointed out in [47] that there are potential modeling problems associated with the calculation of *Ve*, and clearly, it can be estimated only in perfused tissue where there is significant leakage of the contrast agent. This problem means that summary statistics presented in [47] and other studies reflect only perfused tissue with contrast agent leakage. In addition, the authors [47] suggest that the relatively low dynamic sampling duration (6 minutes) will affect *Ve* estimates to some degree, which corroborates our analysis. The connection of estimated *Ve* in gliomas with vessel permeability also helps to explain the correlation between Tofts-derived *Ve* and DP-derived PS in Table 3, which shows a strong statistically significant correlation (0.83, *P* < 0.001) in the mutated IDH group and only a moderate statistically significant correlation (0.44, *P* < 0.001) in the wildtype IDH group. This is because vessels in IDH-wildtype gliomas are leakier than in IDH-mutant gliomas, and the *Ve* estimates in more perfused IDH-wildtype gliomas would be more of balance between vessel leakage and the actual extravascular extracellular space than in IDH-mutant gliomas.

As for the Tofts model, the values of *K*^trans^ and *Ve* in the IDH-mutant group were lower than in the IDH-wildtype group (*P* = 0.013, < 0.001), and no significant difference was observed for *K*ep (*P* = 0.525), where *Ve* attained the best diagnostic performance with AUC 0.79. Tofts-derived *Ve* was strongly correlated with DP-derived *Ve*. Though *K*^trans^ is frequently understood as vessel permeability, the correlation with respect to DP-derived PS was very weak. The moderate performance of *K*^trans^ (AUC = 0.69) could be due to the uncertainty on the physiological interpretation of *K*^trans^, which is assumed as the mixture of the transport of the contrast agent within the vascular space and the transport of the contrast agent exchange between the intravascular space and interstitial space [48]. *K*^trans^ describes only PS when the transport of tracer across the vessel wall is limited by permeability. When blood flow is slow, *K*^trans^ becomes flow dependent. As a result, the measured *K*^trans^ could be confounded by the aforementioned but unknown combination of the implicit processes in tissue microenvironment. Overall, the performance of Tofts is evidently inferior to that of DP.

In our study, a significant difference between IDH-mutant and IDH-wildtype gliomas was found in perfusion parameters. Gliomas with IDH mutations manifested with reduced tissue vascularity, blood flow, and vessel permeability, indicating a hypoxic microenvironment in these tumors, which might restrict the cellular differentiation of oncogenic effects. Mutations in the IDH genes result in disruption of the enzyme's normal catalytic activity and production of 2-hydroxyglutarate (2-HG), an oncometabolite, which leads to genetic and epigenetic dysregulation and subsequent tumorigenesis [49].

A main limitation of this study is that only sample statistics of tumor ROI are taken into account in representing the corresponding patient's disease status, though the calculation of kinetic parameters is carried out over each voxel. As tumor is characterized by heterogeneity, particularly, in apparently low-grade gliomas, approximately 80% of those may contain areas of higher-grade anaplasia [50]; thus, analysis with account of parameter distribution or intervoxel information could be helpful in better understanding the underlying tumor tissue microenvironment. Another limitation is the relatively small sample size, which would influence the confidence interval of the estimated cutoff, sensitivity, and specificity. As IDH mutations were present in 80–90% of grade II and III gliomas [11, 51], it would be interesting to investigate the relationship between histologic subtypes and IDH mutation status and track the tumor evolution, which will be explored in a larger dataset in future. The required sample size in a diagnostic accuracy study has been formulated in [52] as a function of the measure of diagnostic accuracy, conjectured level of accuracy, suspected difference in accuracy between the two imaging techniques, observer variability, and ratio of patients without to patients with the condition, where a table was provided to serve as a guidance on sample size estimation in the design of the diagnostic accuracy study. Alternatively, one might use bootstrapping by repeatedly resampling with replacement from observed data to obtain a sequence of estimates on the diagnostic accuracy and take average as the final estimate [53].

## 5. Conclusions

The distributed parameter model in dynamic contrast-enhanced imaging has been applied to glioma IDH mutation differentiation and compared with the conventional Tofts model. The results turned out that IDH-mutant gliomas were featured by low vascularity (*Vp*), low blood flow, and low vascular permeability, suggesting a tissue microenvironment in IDH-mutant gliomas hostile to the cellular differentiation of oncogenic effects. Consistent with previous DSC studies, tissue vascularity was the most prominent for characterizing glioma IDH mutation, with good potential as imaging surrogate for molecular features of gliomas. Besides that, DCE provided additional information on vessel permeability, which also presents a good diagnostic performance in differentiating IDH-mutant from IDH-wildtype gliomas. On top of that, glioma DCE differed from DCE studies of most other types of solid tumor in the lack of clues on cellular mitotic activity due to the presence of the blood-brain barrier.

## Figures and Tables

**Table 1:** Median value and interquartile range (i.e., range between the 25^th^ and the 75^th^ percentile, in parentheses) of kinetic parameters of two tracer kinetic models in IDH-mutant gliomas and IDH-wildtype gliomas and the results of the Wilcoxon test.

Model	Parameters	IDH-mutant (*n* = 24)	IDH-wildtype (*n* = 31)	*P* value
Tofts	*K* ^trans^ (min^−1^)	0.02 (0.01–0.10)	0.07 (0.04–0.09)	0.013
	*Ve* (mL/100 mL)	0.24 (0.00–8.65)	10.01 (7.86–16.10)	<0.001
	Kep (min^−1^)	0.67 (0.28–70.53)	0.49 (0.35–1.00)	0.525
DP	*F* (mL/min/100 mL)	9.21 (4.70–12.82)	12.48 (10.04–14.56)	0.018
	PS (mL/min/100 mL)	0.21 (0.03–1.52)	3.38 (2.41–5.74)	<0.001
	*E* (%)	1.36 (0.50–10.86)	23.10 (16.05–36.99)	<0.001
	*Ve* (mL/100 mL)	0.25 (0.12–9.91)	9.30 (5.69–15.11)	0.002
	*Vp* (mL/100 mL)	0.21 (0.06–0.44)	1.59 (0.93–2.43)	<0.001

**Table 2:** Diagnostic performance of kinetic parameters of two tracer kinetic models in distinguishing IDH-mutant from IDH-wildtype gliomas.

Model	Parameters	AUC	Cutoff value	Sensitivity (%)	Specificity (%)	Accuracy (%)
Tofts	*K* ^trans^ (min^−1^)	0.69	0.02	50.0	96.8	72.7
	*Ve* (mL/100 mL)	0.79	2.81	66.7	87.1	78.2
	Kep (min^−1^)	0.55	0.40	37.5	67.7	67.3
DP	*F* (mL/min/100 mL)	0.69	6.08	37.5	100.0	63.6
	PS (mL/min/100 mL)	0.81	2.30	83.3	77.4	80.0
	*E* (%)	0.80	5.98	70.8	90.3	81.8
	*Ve* (mL/100 mL)	0.75	2.03	62.5	90.3	78.2
	*Vp* (mL/100 mL)	0.92	0.74	87.5	80.7	83.6

**Table 3:** Results of correlation coefficient between Tofts (horizontal) and DP (vertical) model parameters in IDH-mutant and IDH-wildtype gliomas using the Spearman correlation test, where *P* values were indicated in brackets.

DP\Tofts parameters	*K* ^trans^	*Ve*
Mutant		
*F*	0.29 (0.168)	0.34 (0.105)
*Vp*	0.06 (0.790)	0.78 (<0.001)
*Ve*	−0.21 (0.333)	0.94 (<0.001)
PS	0.00 (0.982)	0.83 (<0.001)
*E*	−0.03 (0.906)	0.90 (<0.001)

Wildtype		
*F*	0.24 (0.194)	0.09 (0.615)
*Vp*	0.07 (0.713)	0.44 (0.013)
*Ve*	−0.23 (0.209)	0.95 (<0.001)
PS	0.05 (0.808)	0.44 (0.014)
*E*	−0.20 (0.288)	0.66 (<0.001)

## Data Availability

The availability of the medical data used in the study is subject to legal and ethical policy on patients' privacy in the People's Republic of China.
